# Radiological and functional outcome in unstable, osteoporotic trochanteric fractures stabilized with dynamic helical hip system

**DOI:** 10.1007/s11751-013-0166-7

**Published:** 2013-07-28

**Authors:** Ram Chander Siwach, Rajesh Rohilla, Roop Singh, Rohit Singla, Sukhbir Singh Sangwan, Paritosh Gogna

**Affiliations:** Department of Orthopaedic Surgery, Paraplegia and Rehabilitation, Pt. B.D. Sharma PGIMS, 9-J/28, Medical Enclave, Rohtak, 124001 Haryana India

**Keywords:** Dynamic helical hip system, Osteoporosis, Unstable fracture, Pertrochanteric fracture, DHS

## Abstract

A dynamic hip screw (DHS) remains the implant of choice for stabilization of trochanteric fractures because of its favourable results and low rate of non-union or hardware failure, but complication rates of the DHS are higher in unstable and osteoporotic trochanteric fractures. The proponents of the dynamic helical hip system (DHHS) report that it has the potential to decrease the cut-out rates in such fractures as helical blade allows compaction in osteoporotic femoral head which in itself improves anchorage. The purpose of the present study was to evaluate the radiological and functional outcome of DHHS in unstable and osteoporotic trochanteric fractures. This was a prospective observational study. The mean age of the 51 patients (24 men and 27 women) was 72.8 years. Fractures were type AO31A2.2 in 28 patients and AO31A2.3 in 23 patients. According to DEXA scans, 41 patients had osteoporosis and 10 patients had osteopenia. Osteoporosis was grade 3 in 36 patients and grade 2 in 15 patients according to Singh’s index. The mean follow-up was 1.84 years. The average sliding of the lag screw was 3.6 mm (range 2–10 mm). The mean operative time was 54.74 (range 48–65) min. The average tip–apex distance was 20.24 mm (range 12–28 mm). All but one fractures united. The average time to union was 13.14 (range 11–24) weeks. There were four mechanical complications namely late helical blade migration (*n* = 1), late medialization of shaft (*n* = 2) and varus collapse with cut through (*n* = 1). No patient was noted to have a plate pull-out. The average Harris hip score was 92.87 (range 76–97). The use of a DHHS for stabilization of unstable(AO31A2), osteoporotic trochanteric fractures in the elderly patients was associated with reliable rates of union and functional outcome and a decreased incidence of screw cut-out and side plate pull-out.

## Introduction

Pertrochanteric fractures are common problems in elderly patients. Operative stabilization permits early mobilization and minimizes complications of prolonged recumbency [1]. Stable pertrochanteric fractures are preferably fixed by sliding hip screws [2–4]. In general, for the treatment of unstable pertrochanteric fractures, two options exist: extramedullary or intramedullary stabilization [5]. Each device has its advantages and disadvantages. The advantage of extramedullary fixation, such as dynamic hip screw (DHS), is the relatively simple, safe and forgiving surgical technique [5]. The DHS remains the implant of choice because of its favourable results and low rates of non-union or hardware failure [2–4], but the complication rates of the DHS are higher in *unstable* pertrochanteric fractures; despite the widespread use of the DHS, cut-out rates of 5–17 % have been reported in the literature [3, 6–8]. The most common mode of failure of a DHS is *cut*-*out* of the lag screw from the femoral head [9, 10] followed by *lift*-*off* of the plate from the femur [3, 4, 11]. Wolfgang et al. [12] reported a 19 % mechanical and technical complication rate with unstable pertrochanteric fractures treated with sliding hip screw device. Moreover, osteoporosis, associated with pertrochanteric fractures in elderly patients, also presents a problem for stable osteosynthesis of the fracture [13].

A number of variations of basic sliding hip screws have been proposed because of such complications in elderly patients with AO31A2, 31A3 type fractures; in these excessive collapse can lead to shortening and hardware failure [5, 13]. One proposal was to improve implant anchorage in the femoral head by the use of a helical blade. The shape of the blade leads to improved rotational stability of the femoral head and neck fragment, which is vital for reducing the risk of cut-out, and may contribute to fewer delayed unions or varus angulation in unstable pertrochanteric fractures [14, 15]. The tip of the blade allows for *compaction* of the bone when it is inserted, which is thought responsible for improving anchorage in femoral head [16, 17]. Another study reported that the DHS with fixed angle locking screws (locking side plate) would reduce the risk of DHS failure and would be particularly useful in patients with osteoporotic bone or for patients with less stable fracture configurations [9]. The dynamic hip helical system (DHHS), designed by AO/ASIF, merges the concept of locking side plate, helical blade and dynamic hip screw. Several biomechanical studies have shown that helical blade has the potential to decrease the cut-out rate [14, 15], but few clinical studies have been reported. The purpose of the present study was to evaluate the radiological and functional outcome in elderly patients with unstable pertrochanteric fractures treated with the DHHS. The main outcome measures of the study were union rate, cut-out, the average sliding of the blade and functional outcome.

## Materials and methods

All patients presenting with unstable pertrochanteric fractures to the authors’ institute, a tertiary level centre, between January 2009 and June 2010 were included in the present prospective study. The study was approved by the Institutional Review Board. The *inclusion criteria* were: (1) age over 50 years, (2) unstable pertrochanteric fracture according to AO classification (Fracture AO31A2), (3) all patients with bone mineral density (*T*-score <−1) and Singh’s index grade ≤3 [18] and (4) a minimum follow-up of 1 year. Patients with reverse oblique fractures (AO31A3), stable fractures (AO31A1), fractures extending into subtrochanteric region and pathological fractures were excluded from the study. Fractures were categorized as stable or unstable on the basis of AO/ASIF classification. Fractures from AO31A1.1 to AO31A2.1 are classified as stable pertrochanteric fractures, and fractures from AO31A2.2 to AO31A3.3 are classified as unstable fractures [14]. Out of 172 pertrochanteric fractures, fifty-one patients with unstable pertrochanteric fractures stabilized with the DHHS met the inclusion criteria. The study included only type AO31A2.2 and type AO31A2.3 fractures. There were 24 men and 27 women with an average age of 72.8 years (range 60–85 years; standard deviation ±6.82 years). The right hip was involved in 18 patients and the left in 33 patients. Forty-six patients had fallen, and 5 patients were injured after road traffic accidents. Anteroposterior and lateral radiographs including the full extent of femur from hip joint to knee joint were obtained. Preoperative radiographs were assessed by three blinded observers not associated with treatment for fracture classification. Fractures were classified using AO/ASIF classification and were type A2.2 in 28 patients and A2.3 in 23 patients. To estimate the bone mineral density (BMD), a DEXA scan of contralateral hip was obtained and the value of *T*-score was noted. The *T*-score was <−2.5 in 41 patients, and 10 patients had a *T*-score between −1 to −2.5. The Singh’s index was assessed from anteroposterior radiographs of the contralateral hip. The Singh’s index was grade 3 in 36 patients and grade 2 in 15 patients. The average time interval from injury to operation was 6 (range 3–10) days.

The helical blade is available in lengths of 65–145 mm with the outer diameter of 12.5 mm. The barrel angle varies from 130° to 150° and measures 25 and 38 mm in length. The 135° DHHS barrel used in this study had a 9-mm long key that engages the blade shaft to prevent rotation and a locking side plate. This is different from the standard DHS, where the screw shaft engages the barrel over its entire length. The procedures were performed by the three senior authors of the study. The implant was fixed as per the recommended technique. The locking side plate in DHHS is a combi-hole design allowing non-locking or locking screws to be used. Initially, one cortical screw was inserted to allow directional compression at fracture site, followed by the insertion of locking screws. In the majority of patients, this practice was followed. For most patients, an indirect reduction was attempted, but no attempt was made to reduce the posteromedial fragment if it required extensive soft tissue dissection for fixation. In all cases, efforts were made to achieve optimum positioning of the tip of the screw in the subchondral bone of the femoral head with a combined tip–apex distance measuring <25 mm on anteroposterior and lateral radiographs. Antibiotic prophylaxis was given as per institutional protocol. Patients were taught and encouraged to do pain-free intermittent quadriceps, hip and knee flexion exercises starting on the second postoperative day. Partial weight bearing was allowed with a walker aid and advanced to as tolerated by the patient with full weight bearing encouraged after 12 weeks.

Patients were followed at 6, 12, 24, 52 and 100 weeks and then once a year until last follow-up. Functional outcomes were assessed using the *Harris hip score* [19]. Union was defined as bridging of three of the four cortices and disappearance of fracture line on the plain radiographs for a patient who was able to bear full weight. Non-union was defined as a fracture that did not heal within six months. Radiological parameters (sliding, screw/blade cut-out, varus/valgus angulation, side plate pull-out) were recorded. The s*liding of helical blade* was determined by measuring the length of the root (*R*) of the blade and that of thread (*T*) on radiograph as reported by Hardy et al. [3].

## Results

**Fig. 1:**
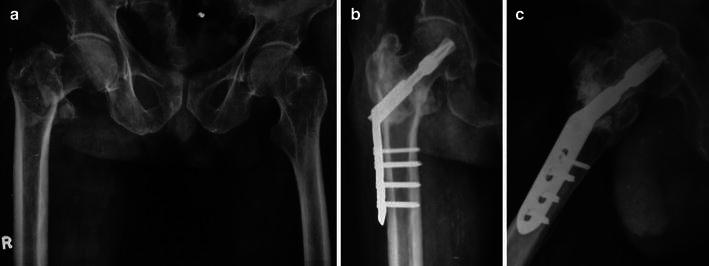
**a** Preoperative anteroposterior radiograph in a 82-year-old male showing 31A2.2 pertrochanteric fracture. **b** Follow-up anteroposterior radiograph of the same patient showing union. **c** Follow-up lateral radiograph of the same patient showing union

**Fig. 2:**
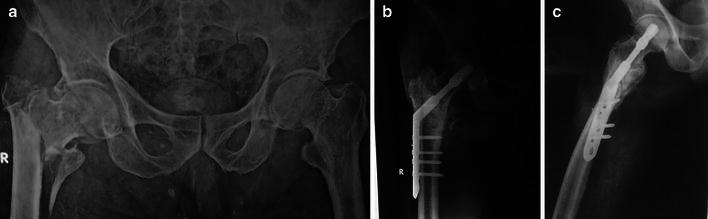
**a** Preoperative anteroposterior radiograph in a 75-year-old male showing 31A2.3 pertrochanteric fracture. **b** Follow-up anteroposterior radiograph of the same patient showing union. **c** Follow-up lateral radiograph of the same patient showing union

The mean operative time was 54.74 (range 48–65) min. The mean follow-up was 20.4 (range 12–28) months. The average sliding of lag screw was 3.6 mm (range 2–10 mm). The average tip–apex distance was 20.24 mm (range 12–28 mm). In two cases, it was more than 25 mm (26 and 28 mm). The average time to union was 13.14 (range 11–24) weeks (Figs. 1, 2). Two fractures had delayed union at 20 and 24 weeks, respectively. One patient had a varus collapse of the fracture. This patient had type AO31A2.3 fracture with grade 3 Singh’s index, but this patient was lost to follow-up. All other fractures healed uneventfully. There were four mechanical complications: late helical blade migration (*n* = 1), late medialization of shaft (*n* = 2) and varus collapse with cut through (*n* = 1) (Table 1). All mechanical complications occurred in different patients. Medialization of shaft was seen at the second month follow-up, weight bearing was delayed in these two cases for 3 months. No patient had side plate pull-out. There were no deep infections or deep venous thromboses. The average Harris hip score was 92.87 (range 76–97). In the final grading as per Harris hip score, 42 patients had excellent results (score 90–100), 6 had good results (score 80–100) and 3 had fair outcome (score 70–80).

**Table 1:** Complications in the present study

Complication	Number of patients (%)
Non-union	1 (2)
Delayed union	2 (4)
Late helical blade migration	1 (2)
Varus collapse	1 (2)
Late medialization of shaft	2 (4)

## Discussion

The best treatment for unstable pertrochanteric fractures remains controversial. The diversity of fixation devices available for treatment of unstable pertrochanteric fractures illustrates the difficulties encountered in the actual treatment. Intramedullary devices have mechanical and biological advantages in such fractures [20]. The dynamic hip screw (DHS) remains the implant of choice because of its favourable results and low rate of non-union or hardware failure [2], but complication rates of the DHS are higher in unstable pertrochanteric fractures; despite the widespread use of the DHS, cut-out rates of 5–17 % have been reported in the literature [3, 6–8]. The DHS is often linked to a high incidence of therapeutic failure in patients with pertrochanteric fractures and a severe degree of osteoporosis [1, 11, 13]. Complications have been associated with cut-out of lag screw from femoral head predominantly, particularly in unstable pertrochanteric fractures [3, 21]. Most mechanical failures involve progressive varus deformity at the fracture site. This may increase tension on the side plate screws, leading to failure of screw–bone interface. The side plate pull-out has been reported in patients with severe osteoporosis [4, 11]. The majority of patients (*n* = 41) in the present study had osteoporosis, and only 4 % patients had fixation failure. No patient had a side plate pull-out in the present study, which may be attributed to the concept of a *locking side plate*. Strauss et al. [15] reported that the biomechanical advantages seen with helical blade fixation of the femoral head compared to sliding hip screw designs may be useful in managing fractures in patients with poor bone quality. We, as a consequence of this review, are also of the opinion that the dynamic helical hip system (DHHS) is a reliable alternative in stabilization of *osteoporotic* pertrochanteric fractures.

In general, for treatment of unstable pertrochanteric fractures, two options exist: extramedullary or intramedullary stabilization [5]. The minimally invasive intramedullary technique is reported to be associated with less blood loss and a lower infection rate; the implant allows early full weight bearing because of its favourable biomechanical properties [5, 19], but screw cut through in 8 % and re-operation in 7.1 % patients have been reported in unstable pertrochanteric fractures treated with proximal femoral nail (Table 2) [20, 22]. Screw cut through was observed in 2 % patients in the present study. Union was achieved in all patients except in one case which was lost to follow-up. Eighty-six per cent in the present study had good to excellent functional outcome with mean Haris hip score of 92.87, which is comparable to average scores (83–90) reported in the literature [23–25]. Barton et al. [21] reported a randomized study comparing long gamma nail and sliding hip screw in treatment of type AO31A2 fractures and concluded sliding hip screw should remain a gold standard for the treatment of such fractures. We report that the DHHS is a reliable alternative for stabilization of *unstable pertrochanteric* fractures. Only two patients in the present study had a tip–apex distance more than 25 mm. The importance of the tip–apex distance is likely to be greater in patients with unstable pertrochanteric fracture [21]. Although the apex–tip distance is originally described for the standard DHS, we have used this method in the present study also to assess the implant position. Reduction in cut-out numbers will not be accomplished by newer implants since implant design cannot make up for suboptimal fracture reduction or poor implant position [5].

**Table 2:** Comparison of studies reporting treatment of unstable trochanteric fractures

	Implant	Average age (years)	Unstable fractures (%)	Operation time (min)	Cut-out (varus) (%)
Hardy et al. [3]	DHS	80	74	57	15
Bucicuto et al. [8]	CHS	81	100	63	15
Adams et al. [4]	SHS	81	53	61	3
Al-yassari et al. [20]	PFN	84	77	–	8
Banan et al. [22]	PFN	79	83	–	8.7
Fitzpatrick et al. [29]	DHHS	80.41	5	34.52	None
Present study	DHHS	72.8	100	54.74	2

Several biomechanical studies have shown an advantage of the helical blade over a screw type implant for unstable and osteoporotic pertrochanteric fractures. Strauss et al. [15] concluded that fixation of the femoral head with a helical blade was biomechanically superior to fixation with a standard sliding hip screw in a cadaveric, unstable pertrochanteric hip fracture model. In a cellular polyurethane foam surrogate model of the femoral head, Sommers et al. [26] demonstrated that the helical blade of the pertrochanteric fixation nail provided the greatest resistance to cut-out compared to the lag screw design of the extramedullary dynamic hip screw and the intramedullary gamma nail. Jewell et al. [9] compared the standard DHS design with a DHS fixed to shaft of femur with locking plate and concluded that a locking screw DHS would be particularly useful in patients with osteoporotic bone and in patients with less stable fracture configurations. Windolf et al. [27] compared the mechanical performance of the DHS and helical blade in paired cadaveric specimens under dynamic loading. They noted 100 % cut-out in the DHS group, but only 50 % cut-out in the helical blade group. They also noted increased fracture collapse in the helical blade group. The compressed bone around the helical blade theoretically provides improved resistance to cut-out relative to the osteoporotic, non-compressed bone surrounding the DHS [17]. Additionally, these spiral blade implants may provide better rotational control of the fracture construct, especially when the lag screw is placed in an eccentric position [26].

Late *helical blade migration* was seen in an otherwise asymptomatic patient at the sixth month of follow-up that had little impact on fracture healing. The tip–apex distance in this case was 16 mm. A similar late migration of tip of the helical blade was reported by Gardner et al. [14] after pertrochanteric fixation nail in elderly patients with pertrochanteric fractures. All position changes occurred within first 6 weeks postoperatively, with no subsequent detectable migration or telescoping with no significant differences between stable and unstable fractures [14]. Gardner et al. also reported reverse migration of blade in 8 % cases and intra-articular penetration in one patient. We did not observe reverse migration of blade in the present study. The average sliding of lag screw was 3.6 mm (range 2–10 mm) in the present study, which was lower than a previous study using the DHS alone [28]. This supports the concept of a fixed angle implant and bone construct with a locking side plate, both of which provides stable fixation in unstable pertrochanteric fracture. Fitzpatrick et al. [29] in their randomized controlled trial have reported the average sliding of 7.4 mm with dynamic helical blade group. Short shaft engagement of helical blade could have led to the binding of the blade in the barrel. This could explain the lesser degree of sliding obtained in the present study.

The literature has few clinical studies evaluating the role of a DHHS in extra-capsular fractures of femur. Fitzpatrick et al. [29] conducted a randomized prospective study on 51 patients comparing the locking helical blade with a dynamic hip screw. They found out no significant difference in the radiographic outcomes of pertrochanteric hip fractures treated with either of these implants. The helical blade group had two failures with central cut through which they relate to a defect in rotational control mechanism. The limitation in their study was that eighty per cent of their fractures were stable in nature (40 out of 51), but the present study included unstable osteoporotic pertrochanteric fractures only.

The present study also has its own limitations; the number of patients is too small to resolve the current controversies. The present study does not have a control group. The surgeries were conducted by surgeons of varied lengths of experience but may have the advantage of the results of the present study applicable to a majority of orthopaedic surgeons performing hip surgery.

## Conclusion

In the present clinical study, the use of a DHHS for stabilization of unstable (AO31A2), osteoporotic pertrochanteric fractures in the elderly patients was associated with reliable rates of union and functional outcome and decreased incidence of screw cut-out and side plate pull-out as compared to standard DHS.
